# Development and Test of Low-Cost Multi-Channel Multi-Frequency Lock-In Amplifier for Health and Environment Sensing

**DOI:** 10.3390/s24186020

**Published:** 2024-09-18

**Authors:** Fabio Pollastrone, Luca Fiorani, Ramanand Bisauriya, Ivano Menicucci, Claudio Ciceroni, Roberto Pizzoferrato

**Affiliations:** 1 Diagnostics and Metrology Laboratory, Physical Technologies and Security Division, Nuclear Department, ENEA (Italian National Agency for New Technologies, Energy and Sustainable Economic Development), Via Enrico Fermi 45, 00044 Frascati, Italy; luca.fiorani@enea.it (L.F.); ivano.menicucci@enea.it (I.M.); claudio.ciceroni@enea.it (C.C.); 2 Department of Industrial Engineering, University of Rome Tor Vergata, 00133 Rome, Italy; r.bisauriya@gmail.com (R.B.); pizzoferrato@uniroma2.it (R.P.)

**Keywords:** lock-in amplifier (LIA), digital signal processing, photoluminescence measurements, microcontroller

## Abstract

Optical-based sensing techniques and instruments, such as fluorometric systems, absorbance-based sensors, and photoacoustic spectrometers, are important tools for detecting food fraud, adulteration, and contamination for health and environmental purposes. All the aforementioned optical equipments generally require one or more low-frequency Lock-In Amplifiers (LIAs) to extract the signal of interest from background noise. In the cited applications, the required LIA frequency is quite low (up to 1 kHz), and this leads to a simplification of the hardware with consequent good results in portability, reduced size, weight, and low-cost characteristics. The present system, called ENEA DSP Box Due, is based on a very inexpensive microcontroller proto-board and can replace four commercial LIAs, resulting in significant savings in both cost and space. Furthermore, it incorporates a dual-channel oscilloscope and a sinusoidal function generator. This article outlines the architecture of the ENEA DSP Box Due, its electrical characterization, and its applications within a project concerning laser techniques for food and water safety.

## 1. Introduction

Optical-based sensing represents a powerful contactless tool to detect and identify low levels of specific analytes such as pollutants or additives in food fraud, adulteration, and contamination. This approach takes advantage of different optical techniques such as fluorimetry and absorbance-based spectrometry. In these cases, the optically produced signals are generally low and affected by bias and different types of noise. Therefore, a strategy to extract the signal of interest is required and can be implemented with LIAs. In general, LIAs perform the quadrature demodulation of a signal with a proper carrier/modulation, removing the effect of all the noise at frequencies different from the carrier. The sinusoidal carrier frequency can lie in very different ranges, depending on the applications of the system. Since 2000, at ENEA C.R. Frascati Laboratories, measurement instruments based on LIA architecture have been developed in the medium/high carrier frequency range for use in laser interferometers [1], RF reflectometers [2], optical radars [3], fluorescence measurements [4], and bolometers [5]. In high-frequency carrier applications, such as optical radar, a Field Programmable Gate Array (FPGA)-based architecture is mandatory. Conversely, in other applications such as photoacoustic spectrometry and fluorescence measurements, the carrier frequency can be very low. In fact, the signal frequency can be chosen within the audio bandwidth (e.g., ~200 Hz); considering this, a sampling and processing rate of 5 ksps (5 k samples per second) is sufficient, being higher than the Nyquist frequency. Some low-cost LIAs implementing different hardware architectures, microcontrollers, and digital processing algorithms have been reported in the literature [6,7,8,9,10]; in our previous implementation [4], we decided to develop a fully digital microcontroller-based architecture.

An evolution of the LIA [4] used in previous years for audio signals has been developed and described hereafter. This new version, called ENEA DSP Box Due, can work on two channels with two modulation frequencies. The main hardware differences from the previous architecture [4] are the presence of a more powerful microcontroller board and a more advanced signal conditioning mezzanine based on the ENEA project and developed by IT Systems, Rome, Italy, similar to other *open-source* instrumentations [6,7]. 

ENEA DSP Box Due was specifically designed for fluorescence-based sensing systems for the detection of adulteration and contamination of food and drinking water. In this regard, heavy metals (HMs) represent one major class of contaminants in aqueous media that continuously accumulate in the environment due to the increasing man-made activity and intrinsic non-biodegradability. In fact, their ubiquitous presence can lead to a wide range of environmental problems and risks to human health [11,12]. For example, Cu(II), is an essential microelement involved in many biological processes such as growth, metabolism, and the immune system [13]. On the other hand, it tends to accumulate in fish (particularly in gills), where it causes a range of diseases and eventually death [11]. More worryingly, in humans, extended exposure to Cu(II) can damage the kidneys and liver and cause gastrointestinal problems and several neurodegenerative syndromes such as Alzheimer’s and Parkinson’s diseases [14]. Many recent investigations have demonstrated that effective sensing of copper in water can be easily achieved through the fluorescence response of carbon dots (CDs), which are carbon-based nanoparticles with peculiar optical properties [15].

This paper describes the DSP Box Due, its characterization with synthetic signals, and the results of its application to fluorescence sensing of copper ions in water using CDs.

## 2. Principles, Materials, and Methods

### 2.1. ENEA DSP Box Due Hardware

**Figure 1:**
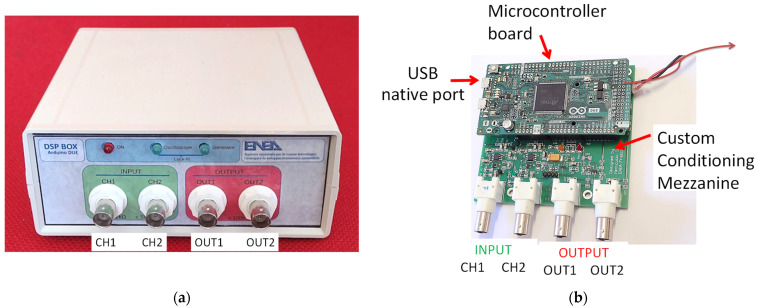
ENEA DSP Box Due pictures. (**a**) Chassis and front connector; (**b**) internal content and subcomponent details.

The ENEA DSP Box Due hardware consists of a microcontroller board and an Enea custom conditioning mezzanine. The technique used to implement the LIA functionality involves fully digital signal processing, similar to the previous development [4]. The input signals are directly sampled and digitally processed by the microcontroller, reducing the problems of non-linearity, parameter variability, and filter implementation associated with analog components (e.g., mixers). The microcontroller board (see Figure 1) is the low-cost Arduino Due [16] based on the 32-bit Atmel SAM3X8E ARM Cortex-M3 CPU microcontroller with an 84 MHz crystal-oscillator, 54 digital input/output pins (12 of which can be used as PWM outputs), 12 × 12 bits of resolution Analog to Digital Converter (ADC), 2 × 12 bits of resolution Digital to Analog Converter (DAC), 4 UARTs (hardware serial ports) and USB OTG capable connection.

The *custom conditioning mezzanine* consists of the following:

Two input channels (CH1 and CH2):

- DC block and a DC offset circuitry necessary to pull up the input signal in case of negative signals, such as in the case of output from Photo Multiplier Tubes (PMTs);
- Anti-aliasing low-pass filters;
- Programmable Gain Amplifier (PGA model MCP6S21), necessary in the case of very low signal, outside the native range of the LIA.
  Two output channels (OUT1 and OUT2):
- Low-pass reconstruction filter;
- Output driver.

### 2.2. Microcontroller Firmware

**Figure 2:**
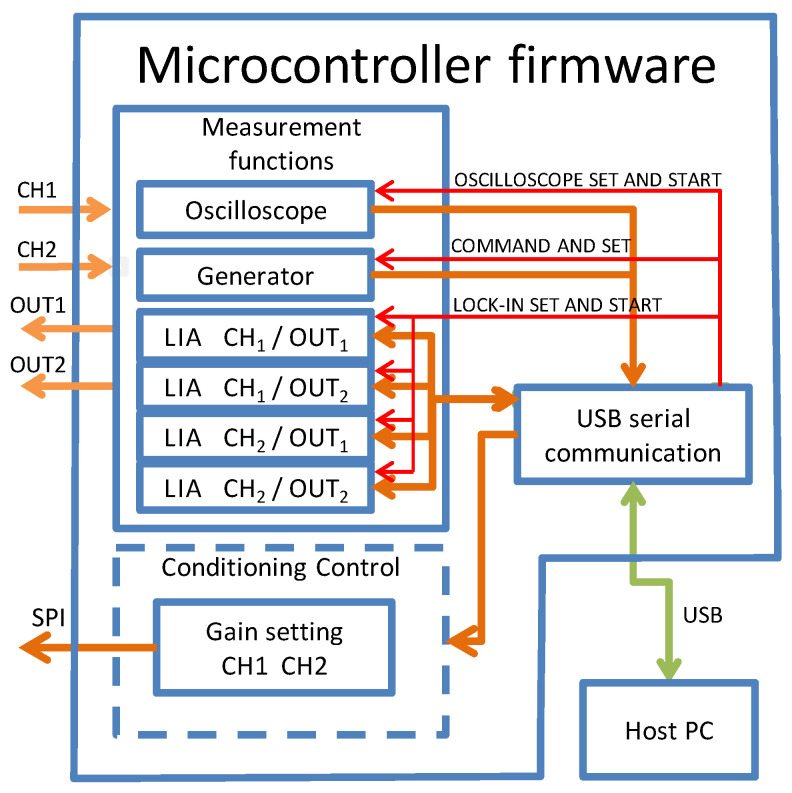
Microcontroller firmware functional diagram.

**Figure 3:**
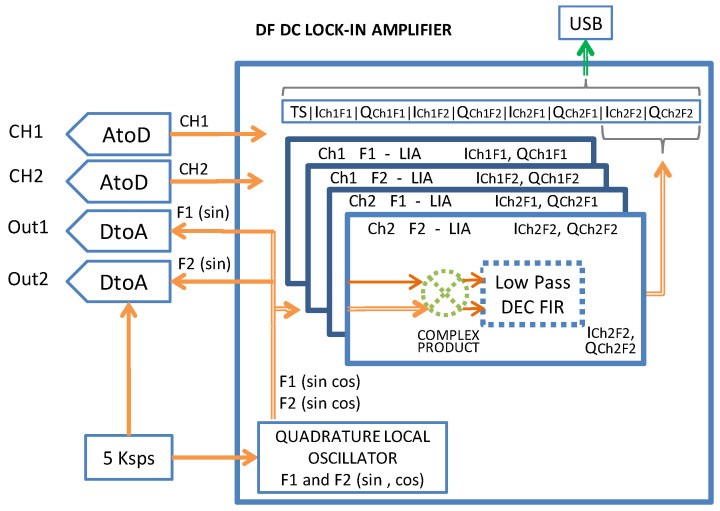
Firmware detail.

The firmware for the microcontroller (see Figure 2 and Figure 3) was developed using the *Arduino IDE* Ver. 1.8.19 in the C language, the native library for trigonometric functions and 16-bit floating-point variables; all this reduced the code development time while slightly increasing the execution time.

The main functions of the firmware are as follows:

 *Communication with the Host PC and PGA setup;*
 *Dual-channel oscilloscope;*
 *Dual-channel generator;*
 *Quad-LIA—frequency setting and measurements.*

See Figure 2.

Details of the functions listed above are given below:

*Communication with the Host PC and PGA setup*

The microcontroller board communicates settings and commands to the *Host PC* via Native (USB3 port) in the serial protocol (250,000 Baud). Generic serial monitor software or the custom GUI described in Section 2.3 must be installed on the *Host PC*. In addition, the microcontroller receives the desired settings for both PGAs from the *Host PC* and sends the SPI instructions to the PGAs on the conditioning mezzanine.

*Dual-channel oscilloscope*

The oscilloscope firmware acquires the CH1 and CH2 signals up to 10 ksps. To guarantee the sampling rate, even in case of low throughput of the USB port, the microcontroller acquires the CH1 and CH2 signals and stores them in an array. When the array is full, the microcontroller stops the acquisition and sends the data to the host PC.

*Dual-channel generator*

The generator firmware generates one sinusoidal signal for each output channel. The output frequency can be in the range DC-2.5 kHz.

*Quad-LIA—frequency setting and measurements*

The DSP Box Due implements a Double Frequency Dual Channel LIA (DF-DC-LIA) algorithm in a digital way, similar to other FPGA-based designs [1,2,3,17], but the decision to use a microcontroller board instead of an FPGA board reduces the cost, the development, and the time required for possible upgrades.

The DF-DC-LIA firmware generates two synthetic sinusoidal signals at frequencies F1 and F2 and a 5 ksps rate on channels OUT1 and OUT2, respectively. These two outputs are the reference signals for the LIAs. The microcontroller firmware implements, for both input channels (CH1 and CH2 sampled at 5 ksps 12 bits) and for both frequencies F1 and F2, a real-time quadrature demodulation with fully digital signal processing (see Figure 3). The single LIA algorithm consists of the digital multiplication of the sampled inputs (CH1, CH2) with the reference signals (sin and cos at frequencies F1 and F2). Each complex product generates two output signals that, when filtered with a low-pass decimation filter, result in four pairs of low-frequency components (I, Q). The rate of the measurements can be changed by varying the decimation rate; typically, for our applications the output frequency is 1 measurement per second. For every measurement, a record containing the time stamp and the four complex components (ICh1F1, QCh1F1, ICh1F2, QCh1F2, ICh2F1, QCh2F1, ICh2F2, and QCh2F2) obtained by demodulating the input signals is composed and then converted into an ASCII string and sent in real-time to the *Host PC* via the USB port.

### 2.3. Host PC Software and User Interface

**Figure 4:**
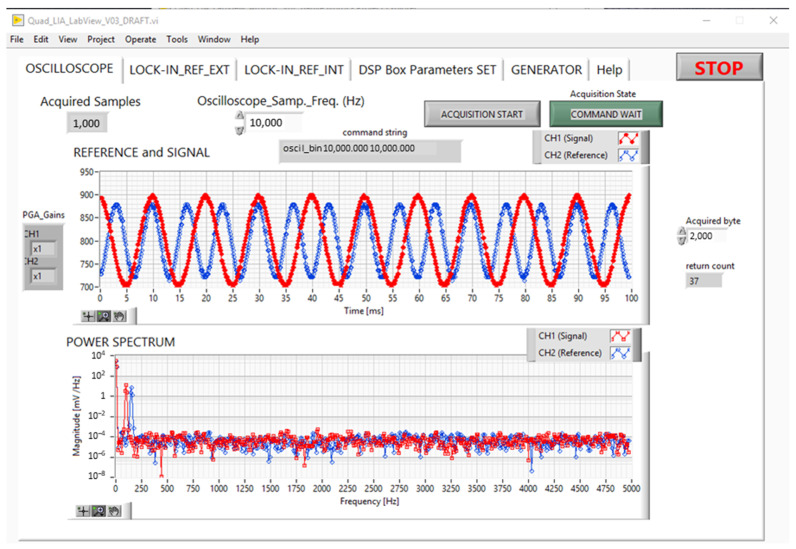
GUI: tab for oscilloscope function. Fs 10 ksps, CH1 200 mVpp 100 Hz, CH2 150 mVpp 150 Hz.

**Figure 5:**
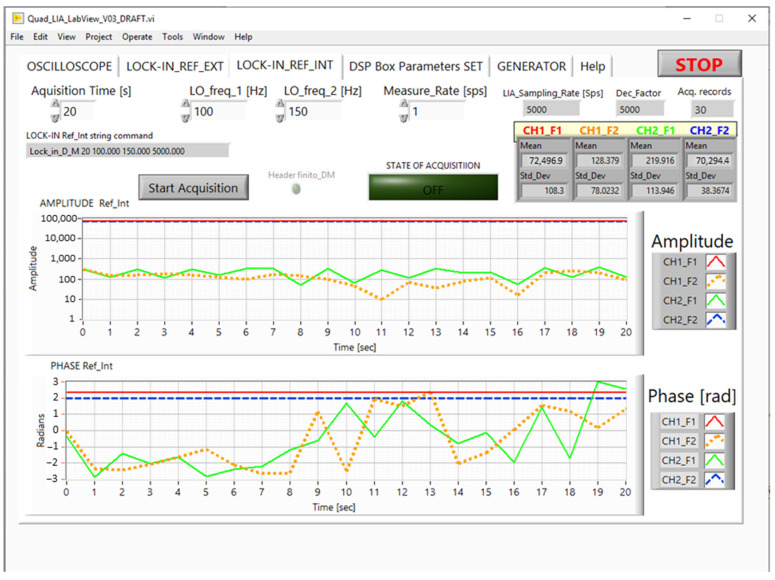
GUI: tab for the Quad-LIA function.

The *Host PC* is connected to the microcontroller board via a USB port in the serial protocol (Figure 1 and Figure 2). The microcontroller receives command strings (e.g., start_oscilloscope <<sampling freq>> or Lock_in_D_M <<Acq time, freq_Out_1 freq_Out_2, output_average) and responds by sending the relative CH1/CH2 acquired data, the Quad-LIA measures, or acknowledges the setup operations to the *Host PC*. It should be noted that the command strings can be sent to the microcontroller in command-line interface mode using generic software. In the present case, an easy-to-use Graphical User Interface (GUI) has been developed in the LabVIEW 2018 environment. Figure 4 and Figure 5 show the GUI for the Oscilloscope and Quad-LIA functions.

#### 2.3.1. Oscilloscope GUI

Although the Quad-LIA is the main function of the DSP Box Due, in order to check the input noise or the amplitude of the signal, an oscilloscope function has been implemented in the DSP Box Due. In particular, the Oscilloscope tab of the GUI turns the DSP Box Due into a two-channel oscilloscope with a sampling frequency of up to 10 Ksps and an input channel with variable gain (1, 2, 4, 5, 8, 10, 16, and 32 *v*/*v*).

The setting for the PGAs gain is located in the “*DSP Box Parameter Set* tab” (see Figure S1 in Supplementary Materials) and is effective for both the *Oscilloscope* and *Quad-LIA* functions.

Figure 4 shows the acquisition of two sinusoidal signals acquired at 10 ksps, with different amplitudes and frequencies CH1 (red curve) 200 mVpp 100 Hz and CH2 (blue curve) 150 mVpp 150 Hz.

#### 2.3.2. Quad-LIA GUI

The Quad-LIA GUI allows you to set the *F1* and *F2* frequencies as well as the *measurement rate* and the *acquisition time*. The *Start_Acquisition* button opens a window to set the measurement file name and to start the measurements according to the processing [18] as explained in Section 2.2. The Host PC receives the records containing the measurements in Cartesian coordinates (I, Q) from the DSP Box Due; the GUI converts this information into polar coordinates (Amplitude, Phase) to facilitate its visualization on the graph.

Figure 5 shows the GUI for the Quad-LIA function; the GUI is similar to the one developed for the DSP Box in the previous project [4], but its graphs show four amplitude and four phase measurements.

In the measurements reported in Figure 5, the input channels CH1 and CH2 are connected to the reference channels OUT1 (100 Hz) and OUT2 (150 Hz), respectively. In this case, the measured amplitudes CH1_F1 and CH2_F2 are at the high level (about 70,000–72,000 a.u.), unlike the amplitude of the crossed values CH1_F2 and CH2_F1 (less than 220 a.u.). In this arrangement, the phase for CH1_F1 and CH2_F2 is fixed, whereas the phase for CH1_F2 and CH2_F1 is almost chaotic, in agreement with theory.

#### 2.3.3. Synthesis of Red-Emitting Carbon Dots

The Red-Emitting Carbon Dots (RECDs) for fluorescence measurements were prepared by using a standard one-step hydrothermal procedure reported earlier [19], with slight modification. Briefly, an aqueous solution of the precursors in a ratio of (citric acid and urea = 1:2) was prepared in DMF (N-N,Dimethylformamide) solvent. Specifically, 4 g citric acid and 8 g urea were dissolved in 40 mL DMF solvent by sonication followed by heating at 180 °C for 4 h in a 100 mL Teflon-lined stainless-steel autoclave. After natural cooling down of the reactor, the obtained product was mixed with 80 mL of ethanol and centrifuged at 4500 rpm for 20 min to wash away the residual solvent and small organic molecules; the washing process was repeated twice.

**Figure 6:**
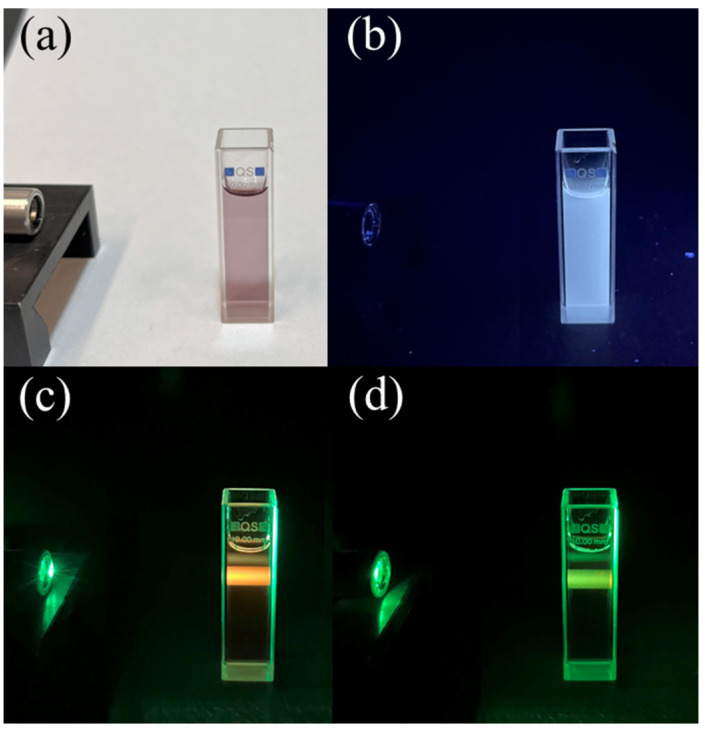
The RECD-sensing solution (**a**) in white light; (**b**) under UV light; (**c**) under 520 nm excitation; and (**d**) under 520 nm excitation after the addition of copper ions at a concentration of 100 μM.

The resulting dark brown colored product had an additional fluorophore in the green and red regions, which were eliminated by dissolving the precipitate in sodium hydroxide solution (50 mg/mL), sonicating for 15 min, and centrifuging at 4500 rpm for 30 min followed by washing with DI water. Afterward, the collected precipitate was further dissolved in hydrochloric solution (5 wt %), sonicated for 15 min, and centrifuged at 4500 rpm for 30 min. The obtained black-colored precipitate was re-dissolved in DI water and centrifuged at 4500 rpm for 30 min 3 times to remove residual hydrochloric acid. The precipitate was dried at room temperature in a desiccator, re-dissolved in DI water, and stored in a freezer at 4 °C as a stock solution for the following experiments. The sensing solution was prepared immediately before the tests by diluting the stock solution in DI water 1:100 in volume and appeared in white light as a transparent liquid with a slight purple color (see Figure 6a). Under UV light, the sensing solution emitted a white fluorescence (Figure 6b), which became red under 520 nm excitation (Figure 6c). Similarly, nitrogen and sulfur co-doped carbon dots (NSCDs) were prepared via a simple one-step hydrothermal method starting from o-phenylenediamine (OPD) and ammonium sulfide [20]. This material was used for two-frequency LIA applications since it changes both its orange fluorescence emission and optical absorbance in the presence of copper ions.

The sensitivity tests in water were carried out as follows: First, 1 mL of de-ionized (DI) water was mixed with 1 mL of RECD-sensing solution by gently stirring for 20 s in order to prepare the reference (blank) solution, which was then used to record the reference fluorescence intensity. Second, the typical sensing experiment was performed in the presence of a heavy metal (HM). This was accomplished by mixing 1 mL of the specific copper solution in DI water at the proper ion concentration with 1 mL of RECD-sensing solution, gently stirring for 20 s, and then recording the sample fluorescence spectrum.

## 3. Experiments and Results

### 3.1. LIA Electrical Testing and Characterization

The main application of the ENEA DSP Box Due is the Quad-LIA functionality. In particular, this functionality can be useful for simultaneous fluorescence and absorbance measurements and for Dial Pass application [10] (not discussed in this article). As explained in Section 3.3, in the fluorescence/absorbance measurements, the outputs of both channels (OUT1/OUT2) are the references for the modulation of two lasers, while the signals coming from one or two photodetectors are connected to the input channels CH1 or CH2 and demodulated with respect to the reference frequencies F1 ≠ F2.

In the previous implementation of a fully digital low-frequency lock-in amplifier [4], the LIA received both Reference and Signal as inputs to the system. In the present version of the ENEA DSP Box Due, the most relevant use of the LIA is within the “*internal reference*” configuration. In this case, the reference sinusoids (synthetically generated by the DSP Box Due and output from OUT1 and OUT2) can be the input for the laser drivers, while the external signals CH1 and CH2 are the input for the DSP.

For this reason, we focused the characterization of the DSP Box Due LIA on the relationship between the measured amplitude and the input amplitude by connecting the generic output OUT1/OUT2 to a resistive voltage divider and sending the output of the voltage divider as an input to channels CH1/CH2.

In this way, it is possible to obtain LIA input signals with different amplitudes but synchronized to the internal reference.

**Figure 7:**
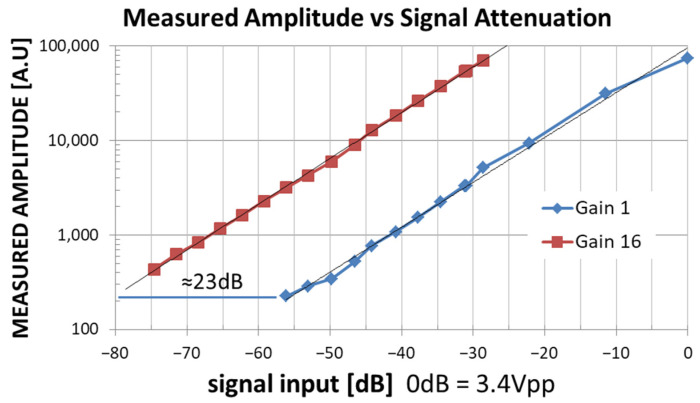
Measured_Amplitude vs. Signal Input level (red curve PGA Gain = 16, Blue curve PGA Gain = 1).

In particular, by varying the amplitude of the input signal, the relationship between the input signal and the measured_amplitude is measured (see Figure 7).

Figure 7 displays the Measured_Amplitude vs. the Signal Input Level at two PGA Gain settings (Gain = 1 blue line and Gain = 16 red line). The horizontal distance between the two interpolating lines is about 23 dB, very close to the theoretical value of 20∙log_10_(∆Gain = 16) = 24 dB. As shown in Figure 7, considering the use of the PGA, the DSP Box Due LIA provides coherent measurements in the input range from 0 dB = 3.4 Vpp to −75 dB ≈ 0.60 mVpp, which can also be considered a good result in relation to the low cost of the DSP Box Due. In fact, in the current version, the input bandwidth of the LIA is limited by the sampling rate to 2.5 kHz, while the maximum measurement output rate is 10 sps; however, this is not a limitation for our applications.

As limits of the study, it should be noted that we have not performed electrical tests by adding white noise to the inputs, like it was done in [4]. Anyway, the digital signal processing used is similar and we checked the bandwidth of the lock-in amplifier, which is less than 0.5 Hz when the LIA is set to 1 measurement per second, in agreement with the theory. This is also necessary to discriminate the effect of the two simultaneous modulation tones (F1 ≠ F2) on the channels CH1 and CH2.

Considering that the system is based on a fully digital signal processing LIA, the presence of noise on the channel, possibly amplified by the PGA from 1 to 32 V/V, does not cause any degradation of the measurement. In any case, it is necessary to take into account the possibility of saturating the *custom conditioning mezzanine* and/or the microcontroller input with the amplified noise. The *oscilloscope* functionality can be useful to verify that the increase in the signal in the input channel (including noise) does not saturate the maximum accepted input level of 3.4 V.

### 3.2. Application to Photoluminescence Measurements: Single-Channel Single-Frequency Mode

**Figure 8:**
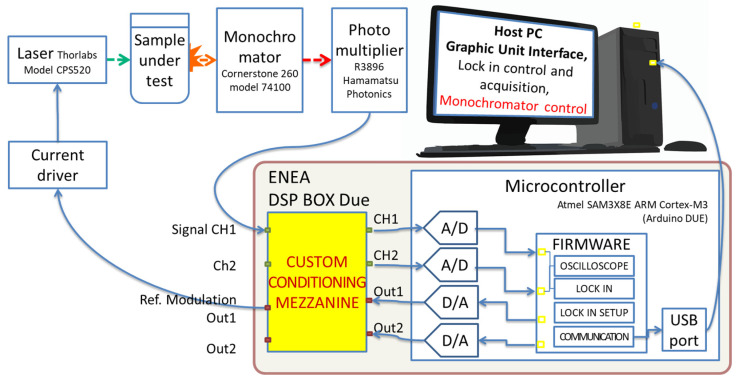
Photoluminescence measurement layout.

The ENEA DSP Box Due was also applied to the detection of the fluorescence signal in a sensing experiment for the presence of heavy metal ions in water. This was performed in a single-channel mode to compare the performance of the DSP Box Due to that of a commercial analog lock-in amplifier (Stanford Research 520) by using a standard laboratory setup [20] for fluorescence measurements. This setup was equipped with a cheap 5V-powered laser diode (CPS520, Thorlabs, Milan, Italy) as a light excitation source at *λ*_exc_. = 520 nm, an emission 25 cm monochromator (Cornerstone 260, Oriel Instruments, Stratford, CT, USA), and an R3896 photomultiplier (Hamamatsu Photonics K.K.) for fluorescence detection (see Figure 8). 

**Figure 9:**
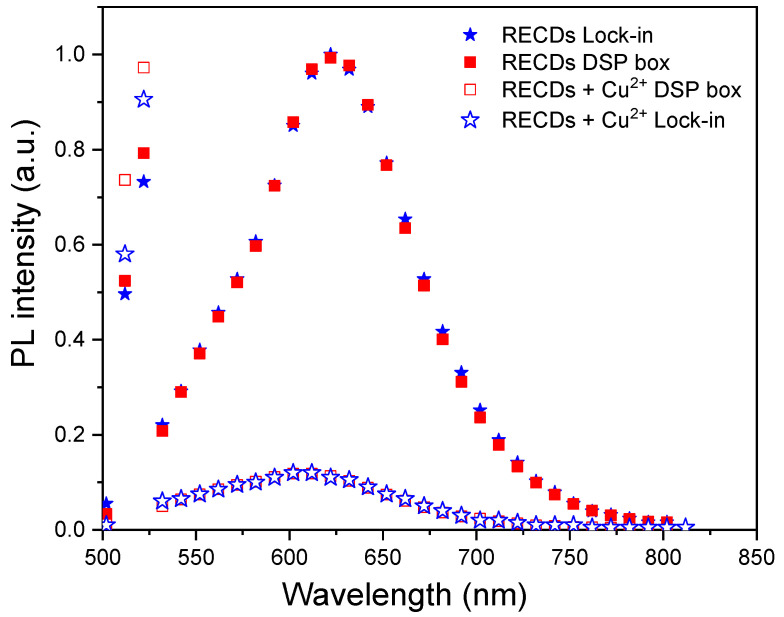
Fluorescence emission spectrum of RECDs excited at 520 nm and measured through a commercial lock-in amplifier (red squares) or by using the ENEA DSP Box Due (blue stars). The empty symbols represent the emission spectrum after the addition of copper ions at a concentration of 100 μM measured through a commercial lock-in amplifier (empty red squares) or by using the ENEA DSP Box Due (empty blue stars).

**Figure 10:**
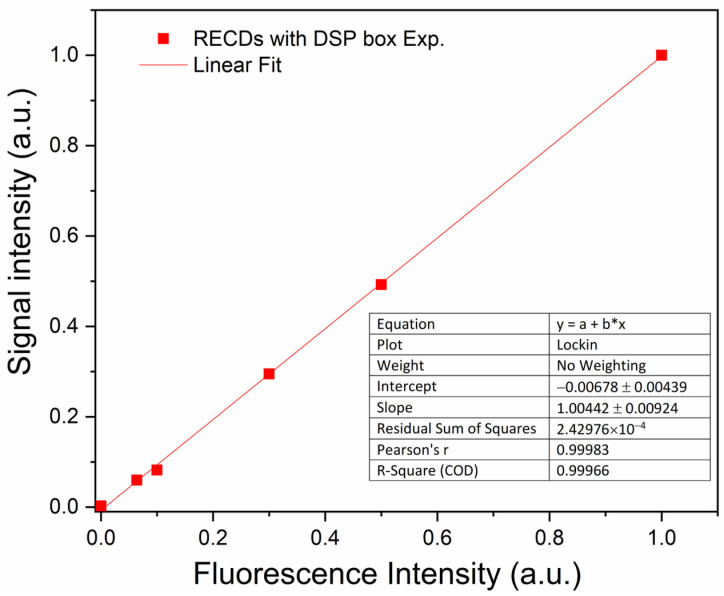
Amplitude of the signal at the output of the ENEA DSP Box Due as a function of fluorescence intensity.

A 2 mL volume of water solution of red-emitting fluorescent carbon dots (RECDs) was used as the sensing medium and held in a fused silica cuvette for excitation by the laser light. The fluorescence emission spectrum of the RECDs is reported in Figure 9 (full red squares) as measured using the DSP Box Due both for the modulation of the laser excitation at a frequency *ν* = 76 Hz and the revelation of the PMT signal with an integration time of 20 s. For comparison, the same spectrum was also measured by revealing the fluorescence signal with the commercial lock-in amplifier after modulation with a mechanical chopper at the same frequency *ν* = 76 Hz as the previous experiment (full blue stars). The two spectra have been normalized to their respective peaks due to the different sensitivities of the two methods. As can be seen, the agreement between the two sets of data is very good, demonstrating the validity of the present system. The linearity of the revelation through the DSP Box Due was tested by varying the fluorescence signal intensity through calibrated optical filters and the results (see Figure 10) showed good linearity of the response with R = 0.9997.

The sensing experiment was performed by adding 1 mL of water containing copper ions (Cu^2+^) to the RECD solution to reach a final concentration of 100 μM Cu^2+^. The interaction and binding of Cu^2+^ with the RECDs produced a fast and remarkable decrease (quenching) in fluorescence emission as shown in the photograph in Figure 6d. This effect was quantified by recording the emission spectrum of the RECD solution before and after the addition of Cu^2+^ (the result is reported in Figure 9). Specifically, the full red squares and blue stars represent the emission spectra of bare RECDs as revealed by the DSP ENEA Box and the commercial lock-in, respectively. On the other hand, the empty squares and stars represent the emission spectra of RECDs after the addition of 100 μM Cu^2+^.

**Figure 11:**
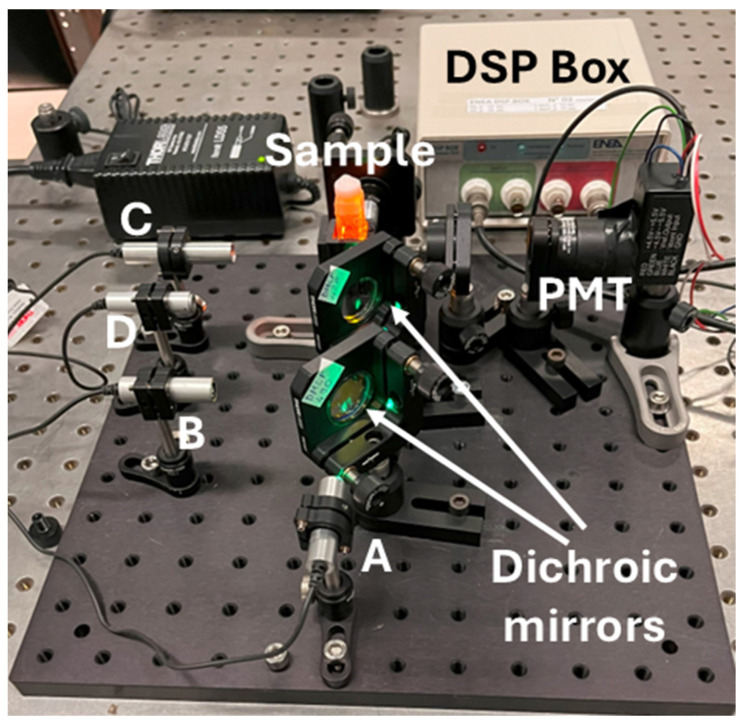
Compact setup for sensing through fluorescence quenching equipped with laser diodes emitting at different wavelengths: (A) 520 nm, (B) 450 nm, (C) 635 nm, (D) 405 nm.

Inspired by these satisfactory results, a simple compact setup for sensing through fluorescence quenching was implemented (see Figure 11) based on the same cheap 5V-supplied laser diode (A) and a 5V-supplied photomultiplier (PMT, H10723-20, Hamamatsu Photonics K.K.). Three additional 5V-powered laser diodes (B, C, and D) with different emission wavelengths were incorporated for further tests, as will be discussed below.

**Figure 12:**
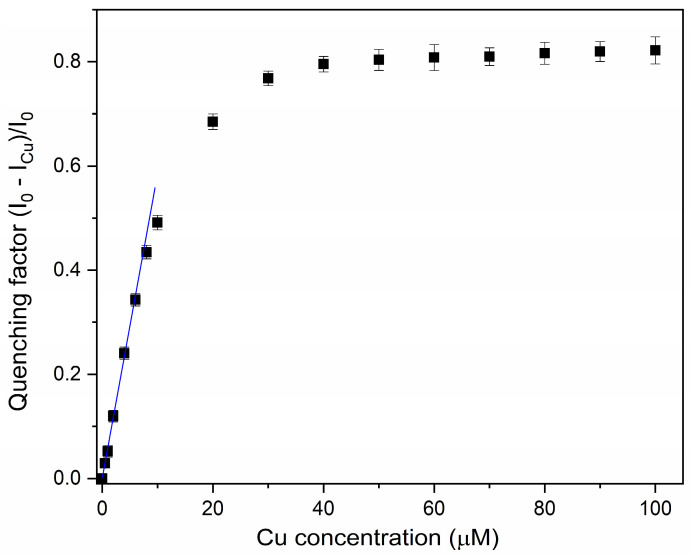
Calibration plot showing fluorescence quenching vs. Cu(II) concentration over the range 0–100 μM. Black squares represent experimental data while the blue line is a guide for the eye showing the linear beahaviour at low concentration. Error bars represent ±SD.

The calibration curve was then recorded using the compact setup by measuring the quenching factor of the RECDs as a function of copper ion concentration (see Figure 12). As can be seen, the response was linear in the range of 0.5–5 μM with a limit of detection (LOD) of 0.6 μM, which is well below the threshold limit of 30 μM set by the WHO guidelines for Cu^2+^.

### 3.3. Application to Photoluminescence Measurements: Two-Channel Two-Frequency Mode

After the promising results with a single-frequency modulation, The ENEA DSP Box Due was also applied to a sensing experiment in a two-channel two-frequency mode with NSCDs as sensing materials. Specifically, by using the compact setup in Figure 11, both the absorbance and fluorescence signals of NSCDs were revealed using the same detector and two light sources at different modulation frequencies. The red-emitting laser B at *λ*_exc_. = 635 nm (see Figure 11 and Figures S2 and S3 in Supplementary Materials) that passed through the sample probed its absorbance and hit the detector was modulated at *ν*_1_ = 190 Hz while the blue-emitting laser C at *λ*_exc_. = 450 nm that crossed the sample at a right angle to excite fluorescence was modulated at *ν*_2_ = 40 Hz. Before entering the sample, laser B was attenuated with appropriate neutral density filters to match the linear range of the detector. It should be stressed that, in this way, the fluorescence signal was measured by the same detector as absorbance. Figure S4 in Supplementary Materials displays the photodetector output that was sent to the two channels of the ENEA DSP Box Due for demodulation. The oscilloscope trace clearly shows the sum of two optical signals at different frequencies, the pseudo-sinusoidal absorbance signal (*ν*_2_ = 3.3 Hz) and the square-wave (*ν*_1_ = 15.5 Hz) fluorescence emission. Two different waveforms were used in this case for the sake of clarity. The operating principle of this method is represented by Video S1 in Supplementary Materials for *ν*_1_ = 10 Hz and *ν*_2_ = 4 Hz.

**Figure 13:**
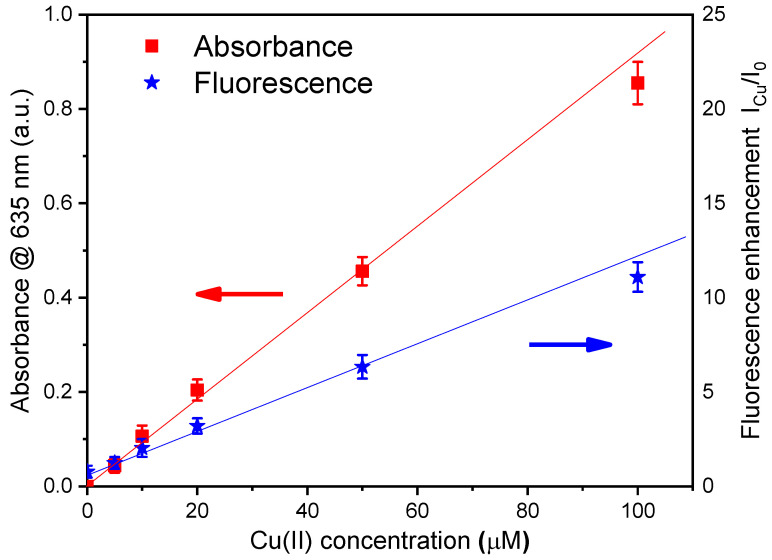
Calibration plot showing fluorescence enhancement (blue stars) and the increase in absorbance (red squares) of NSCDs upon the addition of Cu(II) at different concentrations in the range 0–100 μM. Error bars represent ±SD.

As mentioned in Par. 2.3.3, both the absorbance and fluorescence of NSCDs change in the presence of copper ions [20]. In fact, the two parameters increase linearly with the ion concentration at low levels and this effect is reported in Figure 13 as a function of the Cu(II) concentration over the range 0–100 μM. It should be noted that measuring the simultaneous variations in two different optical parameters as a function of the concentration of a single analyte improves the accuracy of the determination of concentration and reduces the influence of other analytes or effects that might affect the measurement. In addition, this measurement also provided a further comparison of the performance of the ENEA DSP Box Due with a commercial instrument, namely a UV–Vis Cary 50 spectrophotometer (Varian Inc., Palo Alto, CA, USA). As reported in Figure S5, the absorbance data obtained by the ENEA DSP Box compare reasonably well with those recorded by the commercial spectrophotometer and its processing electronics.

## 4. Conclusions

For low-frequency applications, the ENEA Frascati laboratories have developed a new very low-cost DSP Box containing a double channel/double frequency Quad-LIA that can replace four expensive commercial LIAs. The DSP Box Due is based on a microcontroller board (Arduino DUE) and has been electrically characterized, obtaining results compatible with fluorescence sensing applications. The developed system has been tested in sensing applications through fluorescence analysis with good results, similar to those obtained with expensive commercial instruments. We believe that these results will help pave the way for a new generation of simple, portable, and low-cost signal processing circuits and devices for use in a wide range of sensing systems. In particular, the field of wearable or disposable device technologies could benefit from the development of a new class of signal processing systems. Future experiments will include the use of the DSP Box Due in laser photoacoustic spectrometry (LPAS) in relation to new portable LPAS systems designed for Chemical, Bacteriological, Radiological, and Nuclear (CBNR) applications [21]. Possible improvements to the ENEA DSP Box include the development of new hardware based on a more powerful microcontroller to allow signal processing beyond the audio bandwidth.

## Data Availability

No new data are available in addition to those reported in the paper.

## Supplementary Materials

The following supporting information can be downloaded at: https://www.mdpi.com/article/10.3390/s24186020/s1, Figure S1: Screenshot of the “DSP Box Parameter SET” tab of the Quad-LIA-Labview GUI, for diagnostic purposes and for setting the gain of the input channels. Figure S2: The operating principle of the compact sensing system working in two-frequency mode for the simultaneous measurement of both absorbance and fluorescence emission of NSCDs. The red line represents the red-laser beam modulated at *ν*_1_ to probe absorbance. The blue line represents the blue-laser beam modulated at *ν*_2_ to excite the orange fluorescence of NSCDs which is depicted with orange arcs. Figure S3: Signals connections for the compact sensing system operating in two-frequency mode for the simultaneous measurement of both absorbance and fluorescence emission. Figure S4. Oscilloscope trace of the output signal from the photodetector showing the superimposition of the absorbance signal (square wave *ν*_1_ ≃ 15.5 Hz) and the fluorescence signal (pseudo-sinusoidal wave *ν*_2_ ≃ 3.3 Hz). Figure S5: Comparison of the absorbance data of NSCDs upon the addition of Cu(II) at different concentrations in the range 0–100 μM as obtained through the ENEA DSP Box Duo (red squares) and a commercial spectrophotometer (black circles). Error bars represent ±SD. Video S1: The operating principle of the two-frequencies two-wavelengths method.
