# Supplementary material for: Development and Test of Low-Cost Multi-Channel Multi-Frequency Lock-In Amplifier for Health and Environment Sensing
Source: Sensors (Basel). 2024 Sep 18;24(18):6020. doi: 10.3390/s24186020 (PMC11435832; doi:10.3390/s24186020)
Supplement: Supplementary file 1 [file sensors-24-06020-s001.zip › Supplementary Figures S1-S5.pdf]

SUPPLEMENTARY MATERIAL

Development and Test of Low-Cost Multi-Channel Multi-Frequency Lock-In Amplifier for Health and Environment Sensing

Fabio Pollastrone [1,*], Luca Fiorani [1], Ramanand Bisauriya [2], Ivano Menicucci [1], Claudio Ciceroni [1] and Roberto Pizzoferrato [2]

[1] Diagnostics and Metrology Laboratory, Physical Technologies and Security Division, Nuclear Department, ENEA (Italian National Agency for New Technologies, Energy and Sustainable Economic Development), Via Enrico Fermi 45, 00044 Frascati, Italy
[2] Department of Industrial Engineering, University of Rome Tor Vergata, 00133 Rome, Italy
* Correspondence: fabio.pollastrone@enea.it; Tel.: +39-06-9400-5535

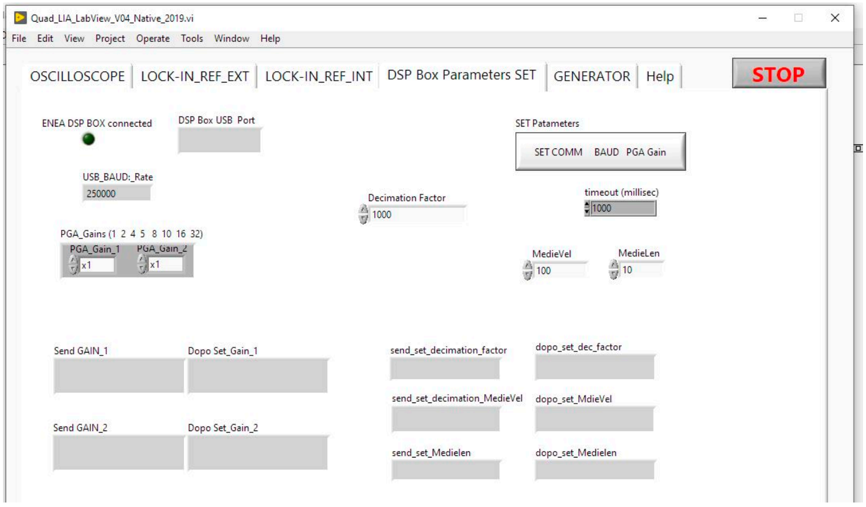

Figure S1. Screenshot of the *"DSP Box Parameter SET"* tab of the *QuadLIA-Labview* GUI, for diagnostic purposes and for setting the gain of the input channels

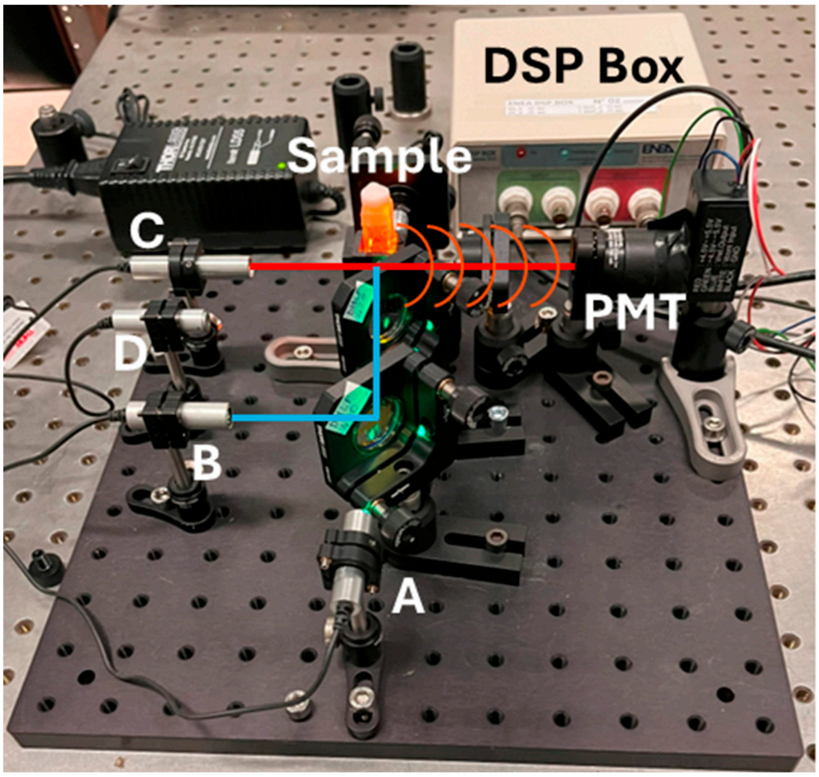

Figure S2. The operating principle of the compact sensing system working in two-frequency mode for the simultaneous measurement of both absorbance and fluorescence emission of NSCDs. The red line represents the red-laser beam modulated at ν_1 to probe absorbance. The blue line represents the blue-laser beam modulated at ν_2 to excite the orange fluorescence of NSCDs which is depicted with orange arcs.

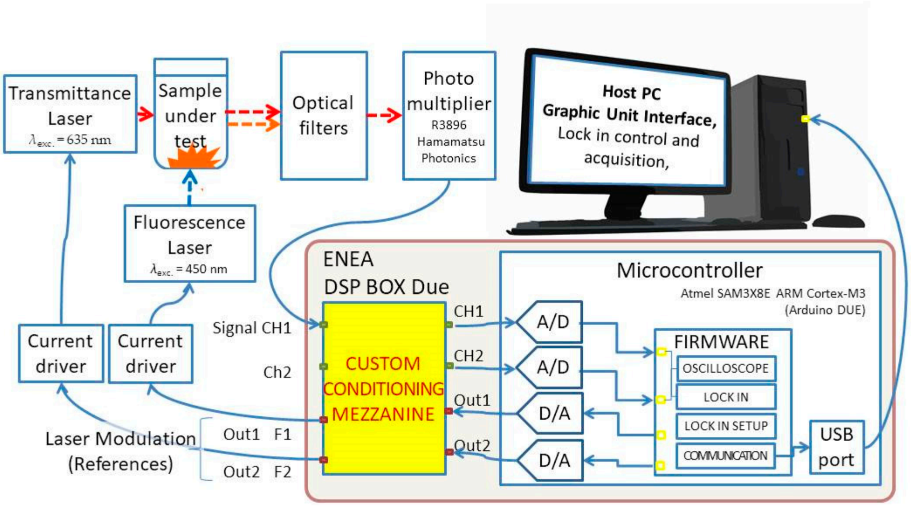

Figure S3. Signals connections for the compact sensing system operating in two-frequency mode for the simultaneous measurement of both absorbance and fluorescence emission.

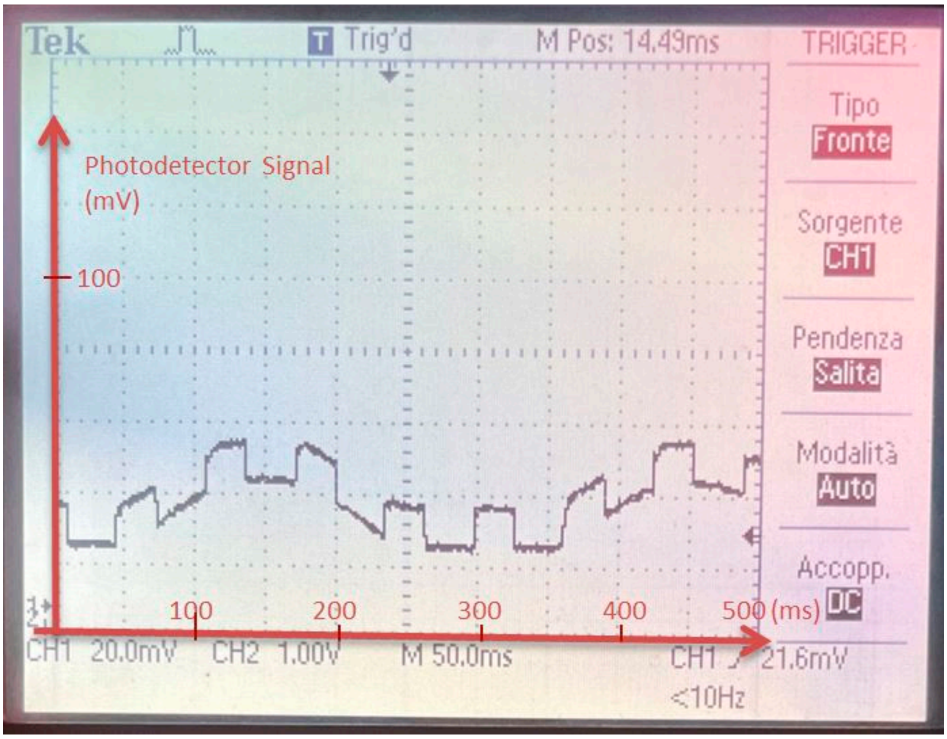

Figure S4. Oscilloscope trace of the output signal from the photodetector showing the superimposition of the absorbance signal (square wave $\nu_1 \simeq 15.5$Hz) and the fluorescence signal (pseudo-sinusoidal wave $\nu_2 \simeq 3.3$Hz).

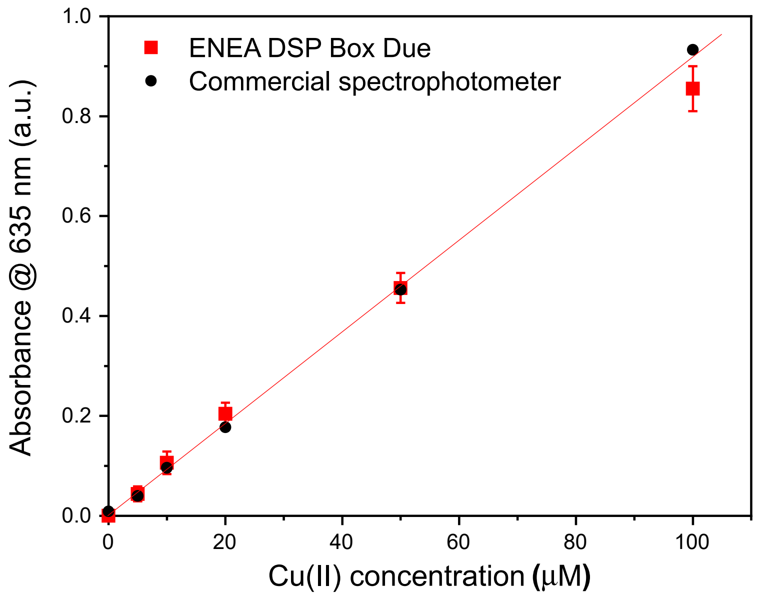

Figure S5. Comparison of the absorbance data of NSCDs upon the addition of Cu(II) at different concentrations in the range 0–100 µM as obtained through the ENEA DSP Box Duo (red squares) and a commercial spectrophotometer (black circles). Error bars represent ±SD.
